# Neuronal Calcium Sensor 1 Has Two Variants with Distinct Calcium Binding Characteristics

**DOI:** 10.1371/journal.pone.0161414

**Published:** 2016-08-30

**Authors:** Baisheng Wang, Göran R. Boeckel, Larry Huynh, Lien Nguyen, Wenxiang Cao, Enrique M. De La Cruz, Edward J. Kaftan, Barbara E. Ehrlich

**Affiliations:** 1 Department of Stomatology, Xiang Ya Hospital, Central South University, Changsha, Hunan, China; 2 Department of Pharmacology, Yale University, New Haven, Connecticut, United States of America; 3 Department of Molecular Biophysics and Biochemistry, Yale University, New Haven, Connecticut, United States of America; 4 Yale Comprehensive Cancer Center, New Haven, Connecticut, United States of America; 5 Department of Cellular and Molecular Physiology, Yale University, New Haven, Connecticut, United States of America; Carl von Ossietzky Universitat Oldenburg, GERMANY

## Abstract

Neuronal calcium sensor-1 (NCS-1 Var1) is a calcium-binding protein expressed in most tissues. We examined a poorly characterized variant of NCS-1 (Var2), identified only in humans where the N-terminal 22 amino acid residues of native NCS-1(MGKSNSKLKPEVVEELTRKTY) were replaced with 4 different residues (MATI). Because alterations in the level of expression of NCS-1 Var1 and the expression of NCS-1 variants have been correlated with several neurological diseases, the relative expression and functional role of NCS-1 Var2 was examined. We found that NCS-1 Var2 mRNA levels are not found in mouse tissues and are expressed at levels ~1000-fold lower than NCS-1 Var1 in three different human cell lines (SHSY5Y, HEK293, MB231). Protein expression of both variants was only identified in cell lines overexpressing exogenous NCS-1 Var2. The calcium binding affinity is ~100 times weaker in purified NCS-1 Var2 than NCS-1 Var1. Because truncation of NCS-1 Var1 has been linked to functional changes in neurons, we determined whether the differing properties of the NCS-1 variants could potentially contribute to the altered cell function. In contrast to previous reports showing that overexpression of NCS-1 Var1 increases calcium-dependent processes, functional differences in cells overexpressing NCS-1 Var2 were undetectable in assays for cell growth, cell death and drug (paclitaxel) potency. Our results suggest that NCS-1 Var1 is the primary functional version of NCS-1.

## Introduction

Neuronal Calcium Sensor 1 (NCS-1; the gene will be abbreviated *NCS1* and the protein will be abbreviated NCS-1) is a high-affinity, low-capacity calcium-binding intracellular protein. A member of the neuronal calcium sensor (NCS) family, NCS-1 contains four helix-loop-helix motifs that are canonical calcium binding domains, usually designated as EF hand motifs. NCS-1 has one N-terminal non-functional pseudo EF hand motif and three functional EF hand motifs that bind calcium [1, 2]. When calcium binds to NCS-1 there are structural changes which, in turn, trigger a cascade of downstream reactions. There also are changes in intracellular calcium that reflect the ability of NCS-1 to both buffer calcium and regulate proteins partners known to bind NCS-1.

The N-terminal region of NCS-1 is a critical region of the protein[3–5]. This is because it contains a myristoylation site which has been proposed to be essential for the control of association with the membrane and proteins in a calcium dependent manner [6] [7]. However, the ability of the myristolyl switch to control membrane association in a calcium dependent manner is debated [8]. Also, the N-terminal region is necessary for proper folding of NCS1 and loss of this region severely attenuates calcium binding to NCS1 [9]. This study examines a naturally occurring variant of NCS1 that is truncated and modified in the N-terminus.

Single nucleotide polymorphisms in NCS-1 are associated with cocaine addiction in African Americans [10] and expression levels of NCS-1 correlate with addiction-like behaviors in rats [11]. Modifications to the expression or mutation of NCS-1 are also associated with schizophrenia [12], bipolar disease [13], autism [14], and chemotherapy-induced peripheral neuropathy [15]. These previous reports have implied that changes in the levels of NCS-1 Var1, and subsequent changes in calcium transients, were responsible for the alterations in cell function. However, the possible expression and role for variants of NCS-1 had not been examined and the primary means of detection of NCS-1 did not distinguish between variants of NCS-1, in particular NCS-1 Var1 and NCS-1 Var2. Therefore, we sought to refine these observations and determine the expression level of NCS-1 Var2 and its potential functional role, assuming it was expressed in human tissues. Also, we know that the N-terminal region, which has the myristoylation site [6] [7], is particularly important for many of the activities associated with NCS-1 function. For example, we have shown that loss of the 36 N-terminal residues of NCS-1 by activation of calpain renders NCS-1 unable to bind calcium in the physiological range [9]. This terminal domain encompasses the region that is altered in NCS-1 Var2.

Generally, increases in NCS-1 result in more release of calcium from the ER [16] and excessive cellular calcium is associated with decreased cell function leading to apoptosis [17]. Elevation of NCS-1 levels were identified in the prefrontal cortex of patients with bipolar disease [13], but the mechanism for the increase in protein and the consequences of these changes are poorly understood. Our previous studies have shown that NCS-1 levels are decreased after chemotherapy [15, 18, 19]. The chemotherapeutic drug paclitaxel binds to NCS-1 and enhances inositol trisphosphate receptor (InsP3R)-dependent calcium signaling [20]. The consequence of the paclitaxel-dependent increase in InsP3R activity leads to augmented cytoplasmic calcium and activation of the class of cysteine proteases called calpains [18]. When calpain is activated, the first 36 amino acids at the N-terminal of NCS-1 are cleaved [9]. The loss of these residues at the N-terminus significantly reduces the calcium binding affinity of NCS-1 [9] and the ability to regulate the InsP3R dependent change in intracellular calcium [21]. These NCS-1 dependent changes in intracellular signaling have been associated with chemotherapy induced peripheral neuropathy [15]. These studies show that NCS-1 plays an important role in signaling sensitivity and intracellular functions.

From an online search (National Center for Biotechnology Information (NCBI) [22] and Ensembl genome browser 83 (doi: 10.1093/nar/gkt1196)), two alternative *NCS1* mRNA-transcripts, *NCS1-002* and *NCS1-003*, processed from the human *NCS1* gene were identified. *NCS1-001* will be designated here as variant 1 of *NCS1* (*NCS1* Var1) and *NCS1-003*, will be designated here as variant 2 of *NCS1 (NCS1* Var2) and both code for protein. In contrast, *NCS1-002* is a non-coding transcript, which means no protein was available to be included in the study. We were particularly interested in NCS-1 Var2 that differs from NCS-1 Var1 by replacing the first 22 N-terminal amino acids of NCS-1 Var1 (MGKSNSKLKPEVVEELTRKTY) with 4 different residues (MATI). This change in the N-terminus of NCS-1 makes modifications that are close to the myristoyl switch site, as identified previously (compare the NCS-1 Var2 site with Fig 1 of ref [8]. Also, the calcium binding is diminished with loss of 36 amino acids from the N-terminal region of NCS-1 Var1, even though calcium does not bind in this region [9]. The proximity of these 2 critical regions in the N-terminus of NCS-1 suggests major functional differences between the two variants. First, there is no myristoylation site on Var2 so membrane association of Var2 should be dramatically less and calcium independent. Second, we would expect calcium binding to be decreased as reported for the calpain-cleaved NCS-1 Var1 [9]. Therefore, we hypothesized that NCS-1 Var2 would display functional properties distinct from NCS-1 Var1.

To test our hypothesis, we compared the two NCS-1 variants. We investigated biochemical properties, including calcium binding, protein conformation and degradation, and we assessed the relative expression of the variants in cell lines and organ tissues. To investigate functional differences, human cell lines were stably transfected with the variants and cell growth rate and the response to paclitaxel were monitored. As expected, NCS-1 Var2 had a reduced calcium binding affinity when compared to NCS-1 Var1. However, the abundance of NCS-1 Var2 was modest when measured as either the mRNA or protein level. Also, no significant functional differences were identified in the comparison of NCS-1 Var1 and NCS-1 Var2. Our results suggest that only one variant of NCS-1 is critical for cell function.

## Experimental Procedures

### Production of NCS-1 variant proteins

**Fig 1:**
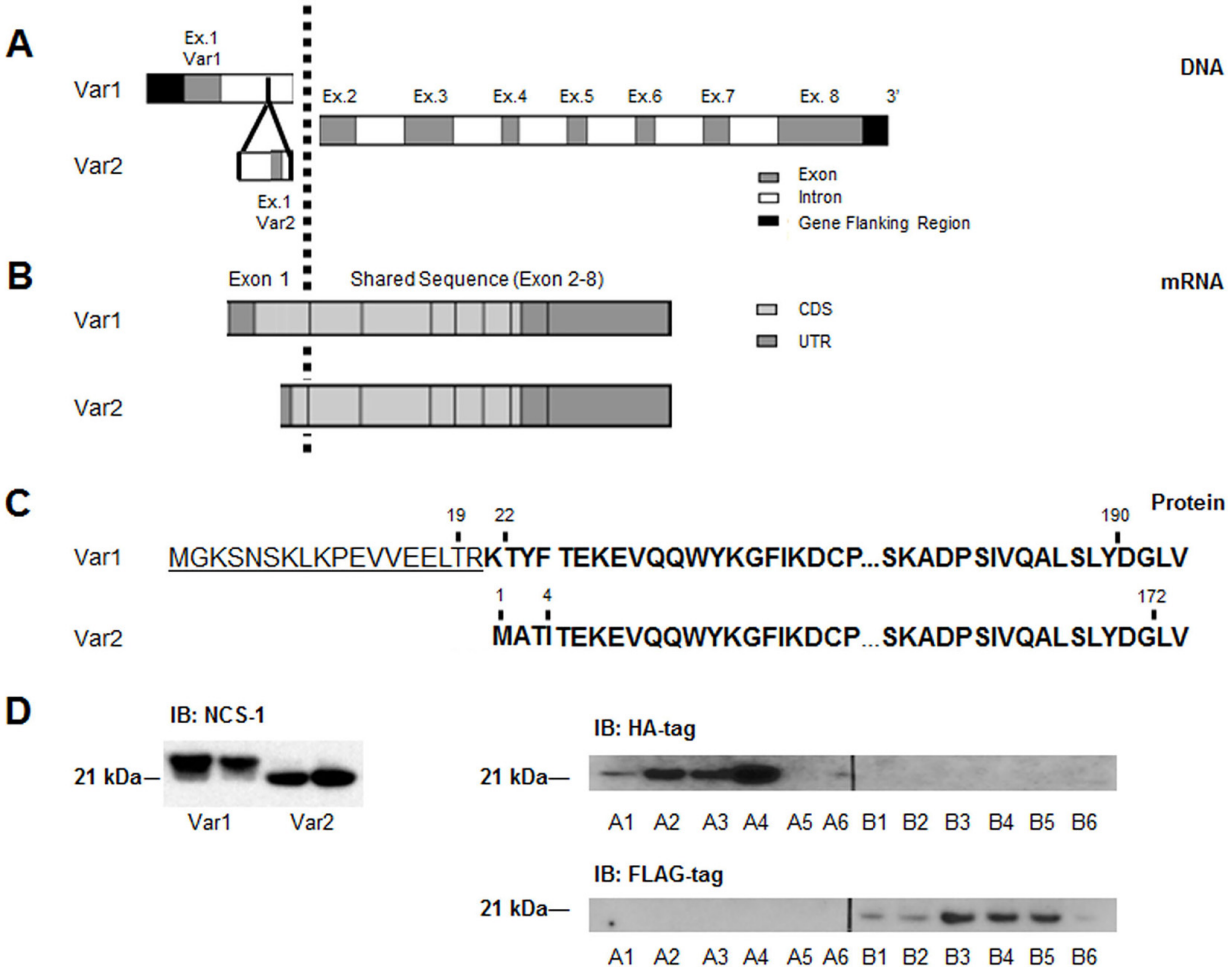
Comparison of NCS-1 variants. (A) The human *NCS1* gene lies on chromosome 9 and consists of 8 exons which code for a 190aa high-affinity, low capacity, calcium binding protein. The NCBI and ensemble databases describe an alternative exon 1, coding for an alternative mRNA towards the 3’ end of intron 1–2 (variant 1) (specific locations being NCS1-001 = 9:130172578–130237304 and NCS1-003 = 9:130200432–130233375) (B) Schematic alignment of the two mRNA variants of *NCS1*. Note that exon 1 of NCS-1 Var2 is significantly shorter than exon 1 in NCS-1 Var1 (19bp vs. 150bp). Around 50% of exon 1 and 80% of exon 7 together with exon 2–6 are part of the coding sequence (CDS) whereas exon 8 is not translated. (C) Schematic alignment of the protein sequence in one letter code. The 22 N-terminal amino acids of NCS-1 Var1 are substituted for four amino acids in NCS-1 Var2. (D) Western blots of SHSY5Y transiently transfected with a vector expressing NCS-1 Var1 or NCS-1 Var2. Left panel probed with FL190, an anti-NCS-1 polyclonal antibody. Right panel probed with anti-HA antibody (top, NCS-1 Var1 is HA-tagged) or anti-FLAG antibody (bottom, NCS-1 Var2 is FLAG-tagged). A1-6 and B1-6 each denote six different clones of SHSY5Y cells transfected with HA-tagged NCS-1 Var1 or FLAG-tagged NCS-1 Var2, respectively.

The plasmid for rat NCS-1 Var1 with a HA tag [9] was used as the template for removing the first 18 amino acid and replacing them with 4 different residues (Fig 1) and a different tag to create NCS-1 Var2 with a FLAG tag (Fig 1D). The human and rat amino acid sequences of NCS-1 Var1 are identical. All plasmids were verified by sequencing.

NCS-1 Var1 and NCS-1 Var2 protein were produced after plasmids were subcloned into pET21-a+, bacterial expression vector. NCS-1 purification protocol was modified from a previous report [23]. The NCS-1 vectors were transformed into Stratagene BL21(DE3) Codon Plus RIL competent E. coli cells. Cells were grown at 37°C in 2 L baffled flasks with 1 L LB broth and ampicillin (100 ug/mL) and chloramphenicol (35 ug/mL). At an OD595nm of 0.5–0.7, overexpression was induced with 1.0 mM isopropyl-d-thiogalactoside (IPTG) and samples were shifted to 18°C for ∼16 h. Cells were harvested by centrifugation (3000 rpm, 30 min, 4°C) and resuspended in 10 mL of 50 mM HEPES, pH 7.5, 100 mM KCl, 1 mM *tris*(2-carboxyethyl)phosphine (TCEP), 1 mM MgCl_2_, and 1mM CaCl_2_.

**Fig 2:**
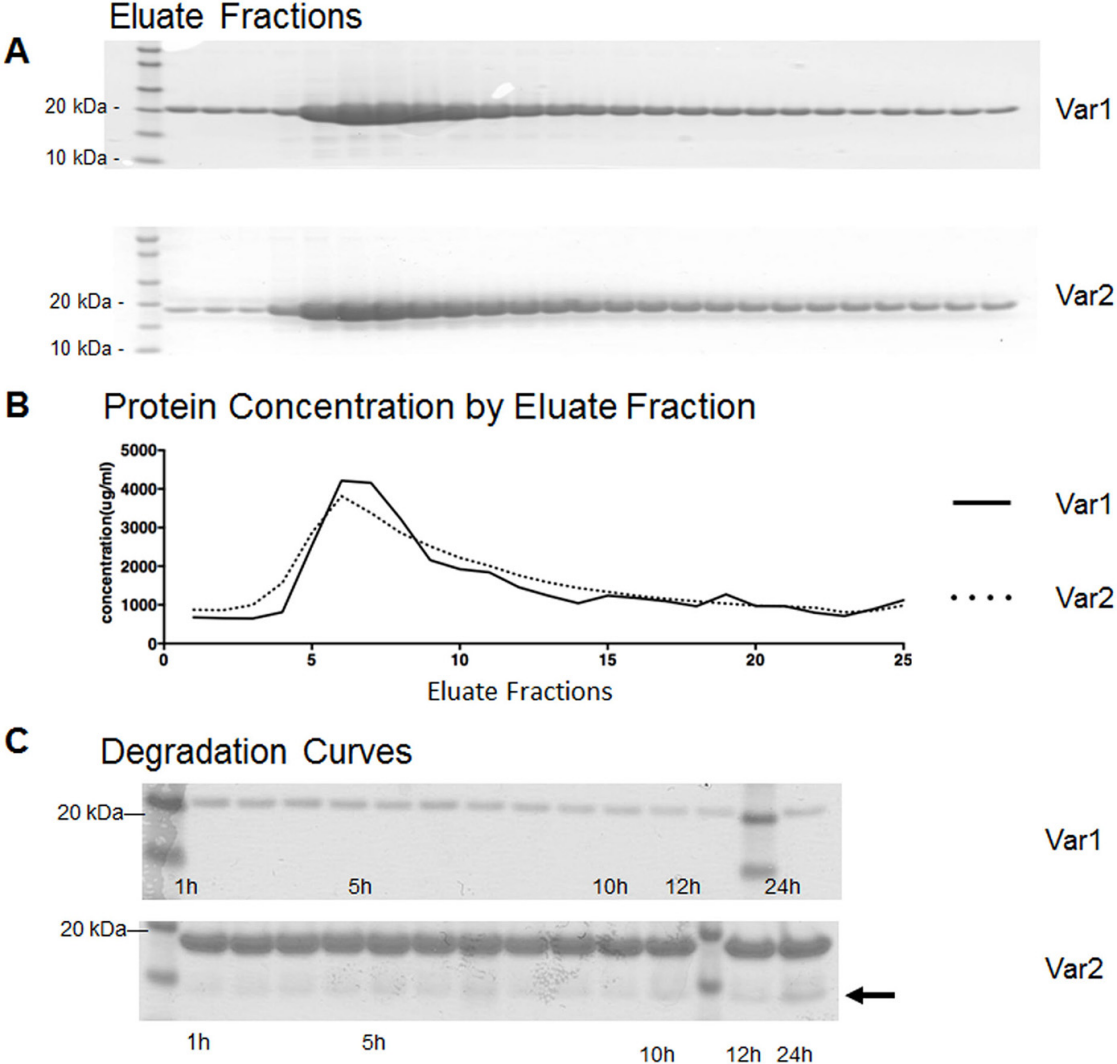
Protein purification and degradation curves of NCS-1 variants. (A) Representative Coomassie blue stained gels showing the purification of NCS-1 Var1 (top) and NCS-1 Var2 (bottom). Far left lane has protein standards. Lanes 2–5 (from left) show flow through. (B) Assessment of protein concentration in each elution fraction. (C) Purified NCS-1 Var1 and NCS-1 Var2 were incubated at room temperature to analyze protein stability. Both isoforms are stable for at least 12 hours. The lanes with 2 bands (near 0 and 12 hour lanes) show molecular weight markers. The arrow on the right of the lower panel shows that there are signs of degradation of NCS-1 Var2 after 24 hours.

Bacteria expressing recombinant NCS-1 were lysed with lysozyme (2mg/mL, Sigma-Aldrich, USA) coupled with 3 freeze–thaw cycles using ethanol-dry ice. Cell lysate was homogenized by tip sonication (Branson Ultrasonics, Danbury, CT) for 2 min on ice using a 50% duty cycle. Homogenized lysate was clarified by centrifugation at 40,000 × g (20,000 rpm, 1 h, 4°C) and the resulting supernatant further sonicated for 2min at 50% duty cycle to reduce sample viscosity. Hydrophobic interaction chromatography (HIC) was used to purify NCS-1 as described previously 4. Large-scale purification of NCS-1 protein was performed using a HiTrap Phenyl HP high substitution 5 mL column (GE Healthcare, USA). The lysates were loaded on a column equilibrated in 50 mM HEPES, pH 7.5, 100 mM KCl, 1 mM TCEP, 1 mM MgCl_2_, and 10 mM CaCl_2_. Following sample application the column was washed with 10 column volumes of 50mM HEPES, pH 7.5, 100 mM KCl, 1 mM TCEP, 1 mM MgCl_2_, and 10 mM CaCl_2_. Recombinant NCS-1 protein was eluted using 50 mM HEPES, pH 7.5, 100 mM KCl, 1 mM TCEP, 1 mM MgCl_2_, and 50 mM EDTA in 25× 1 mL fractions into tubes maintained in a 4°C cold room. The protein was eluted with 50 mM EDTA to improve yield. A cocktail of protease inhibitors was included in all steps during protein purification. Protein purification was monitored using SDS-PAGE. 1 mL elution fractions were collected (fractions 5–7 of 25 mL) and pooled (Fig 2A). The concentration of NCS-1 containing fractions (typically fractions 5–7 of 25 mL) was determined using the BCA Protein Assay (Pierce ThermoFisher Scientific, Waltham, MA). Yields of 10–15 mg of pure NCS-1 were obtained using the above procedure (Fig 2B). Protein was stable when maintained at room temperature for 24 h (Fig 2C).

To prepare calcium-free protein, purified NCS-1 was stripped of calcium as previously described [9]. Briefly, an Econo-Pac 10DG column (Bio-Rad Laboratories, Hercules, CA) was used to exchange the buffer of three 1-mL purified NCS-1 fractions to 50 mM HEPES and 100 mM KCl at pH 7.5. NCS-1 was then dialyzed against 1 L 10 mM EDTA at pH 2.0 using a Slide-A-Lyzer 7K MWCO dialysis cassette (Pierce Thermo Fisher, Waltham, MA) for 1.5 hours, followed by dialysis against 1 L deionized water, followed by dialysis against 1 L 10 mM HEPES pH 7.4 for 1.5 hours. Finally, the protein was dialyzed against 50 mM HEPES, 100 mM KCl, and 0.5 mM TCEP at pH 7.2 overnight. Dialysis was performed using plastic containers. The protein was then concentrated up to 100 uM using an Ultracel 10 K Amicon Ultra-15 centrifugal filter device (EMD Millipore, Billerica, MA).

### Western blot analysis of NCS-1 Var1 and NCS-1 Var2 protein

Cells were lysed as described previously [9], and immunoblotting was performed using the NuPAGE system (Invitrogen, Carlsbad, CA) and the Biorad wet transfer system (Bio-Rad Laboratories, Hercules, CA) according to the manufacturers’ protocols. For these studies three different human cell lines (SHSY5Y, HEK293, MB231) and C57BL/6 mouse tissues were used. Antibodies used were anti-NCS-1 FL-190, anti-HA (both from Santa Cruz Biotechnology, Santa Cruz, CA), and anti-FLAG (Sigma-Aldrich, USA). Protein expression was quantified by scanning densitometry by UN-SCAN-IT (Silk Scientific, Orem, UT) and normalized to β-actin loading controls. All Western blotting experiments were performed using three independent cultures.

### Calcium titration by Isothermal Titration Calorimetry (ITC)

Calcium titrations were performed using a Nano ITC Low Volume machine (TA Instruments, New Castle, DE). The protein sample was exchanged into ITC buffer, 50 mM HEPES, 100 mM KCl, and 0.5 mM TCEP at pH 7.2 using a Slide-A-Lyzer 7K MWCO dialysis cassette (Pierce Thermo Fisher, Waltham, MA). A solution of 1.5 mM calcium CaCl_2_ (in ITC buffer) was titrated into 100 uM calcium-free NCS-1 over thirty-three 1.5 μL injections at 25°C. As a control, calcium ligand solution was injected into buffer solution without protein under the same experimental conditions and resulted in a flat isotherm with negligible heat evolution. Data were processed using the NanoAnalyze software (TA Instruments, New Castle, DE), and binding isotherms were fit using an independent binding site model to determine the dissociation constant (*K*_d_), and the observed enthalpy (Δ*H*°′_obs_), and entropy (Δ*S*°′_obs_) changes associated with calcium binding.

### Circular Dichroism measurements

Purified NCS-1 Var1 and Var2 were analyzed by circular dichroism to test if their structures differed significantly upon calcium binding. Samples of 2 μM calcium-free NCS-1 Var1 or Var2 were prepared in a buffer of 50 mM HEPES, 100 mM KCl, and 0.5 mM TCEP at pH 7.2. Under the calcium-binding conditions, the buffer contained 600 μM calcium. Samples were placed in a 700 μL Quartz SUPRASIL cuvette (Hellma Analytics, Müllheim, Germany) with a path length of 2 mm and measured in a Chirascan CD Spectrometer (Applied Photophysics Limited, Leatherhead, UK). Spectra were averaged over two measurements with their solvent baselines subtracted and analyzed using the K2D [21] analysis from DichroWeb [24].

### Relative expression of NCS-1 variants assessed by PCR

Primers were designed using the NCBI Primer-BLAST software and were purchased from Yale Keck Foundation—Oligo Synthesis Resource. NCS-1 Var1 cDNA region spanning nucleotides 87–273 was amplified with primers V1F (5’-atg ggg aaa tcc aac agc aag ttg aag ccc gaa g-3’) and V1R (5’-tg gtg ggg tct ccg aac ggg-3’) and the NCS-1 Var2 cDNA region spanning nucleotides 3–196 was amplified with primers V2F (5’ -T TGA GAG ATG GCA ACG ATT ACC GAG-3’) and V2R (5’-CAA TTC GCC CGT CCT TGT TTT C-3’) using cDNA prepared from RNA extracted from SHSY5Y, HEK 293 and MB 231 cell lines. The PCR products were cloned into pCR2.1-TOPO vectors according to TOPO TA Cloning Kit protocol (Invitrogen, Carlsbad, CA). All transformations were done using E. coli DH5alpha, grown in LB medium at 37°C. Selection was performed through selection plates containing LB with 100ug/mL ampicillin.

Amplification reactions for the SYBR Green assays contained 2× SYBR Select Master Mix (Applied Biosystems, Foster City, CA), 100 nM of each primer. The thermocycling conditions were 50°C for 2 min, 95°C for 15 min followed by 40 cycles of 94°C for 30 s, 59°C for 40s and 72°C for 40s. Melting curves were generated after amplification. Data were collected using the 7500 Fast & 7500 Real-Time PCR System (Applied Biosystems, Foster City, CA). Each sample was tested in triplicates. To rule out unspecific amplification of genomic DNA samples were run on an agarose gel to show a single amplified band.

For NCS-1 Var1 and NCS-1 Var2 plasmid standard curves, 5-fold serial dilutions were made starting from a plasmid concentration of 5,000,000 copies/ well. The range of each standard curve spanned six points. Concentrations of the plasmids were measured using a UV-Vis Spectrophotometer NanoDrop 2000c (NanoDrop Products, Wilmington, DE). Conversion of nanograms to copy number was done using the formula: number of copies = (amount in ng * 6.022x10^23) / (length in bp * 1x10^9 * 650) under the assumption that the average weight of a base pair (bp) is 650 Da.

### Stable expression of NCS-1 variants in SHSY5Y cells

The optimal concentration of Geneticin (G418) for selection of stably expressing cell lines was determined to be 1mg/ml G418. Passage14 SHSY5Y cells were transfected with NCS-1 Var1 or NCS-1 Var2 plasmid using Lipofectamine 2000 (Thermo Fisher Scientific, Waltham, MA). After 24–48 hours of transfection, cells were split to 1:10, 1:20 or 1:50 into 15 cm plates containing 25 ml of DMEM and 10% FBS and G418. After 4 weeks, isolated colonies begin to appear. Picked colonies were grown and protein expression was checked by Western blot analysis.

### Assessment of proliferation and drug sensitivity in stably transfected cells

Stably transfected SHSY5Y cells were counted and resuspended in culture medium at 1 x 10^5^ cells/mL. Cells were added to a 96-well plate and monitored in an IncuCyte (Essen BioScience, Ann Arbor, MI) incubator at 37°C in 5% CO_2_. Four pictures were taken of each well every 2 h and analyzed for confluence according to the manufacturers’ instructions. To monitor the sensitivity to paclitaxel, cells were resuspended in a range of drug concentrations (per 100 μL: 0ng, 30ng, 70ng, 100ng, 300ng, 500ng) and monitored as for proliferation.

## Results

### NCS-1 variants can be identified and expressed

Alternative *NCS1* mRNA-transcripts were identified with online databases (National Center for Biotechnology Information (NCBI) [22] and Ensembl genome browser 83 (doi: 10.1093/nar/gkt1196)). The variants that code for protein were included in this study. These variants are *NCS1-001*, designated here as variant 1 of *NCS1* (*NCS1* Var1), and *NCS1-003*, designated here as variant 2 of *NCS1 (NCS1* Var2). Both protein coding mRNA transcripts consist of 8 exons of which exons 2–7 are identical (Fig 1A). Exon 1 of *NCS1* Var2 is located towards the 3’ end of Intron 1–2 (*NCS1* Var1; Fig 1A) and is significantly shorter than exon 1 of *NCS1* Var1 (19bp vs. 150bp).

Approximately 50% of exon 1 and 80% of exon 7 together with exon 2–6 are part of the coding sequence (CDS) and exon 8 is not translated at all. For *NCS1* Var2, the codon overlapping junction between exon 1 and 2 results in the translation of distinct amino acids. Essentially, protein sequence alignments shows that in *NCS1* Var2 (NCS-1-003) the N-terminal 22 amino acids of *NCS1* Var1 (NCS-1-001) are substituted for 4 different amino acids (Fig 1C).

After cloning *NCS1* Var1 and *NCS1* Var2 into the mammalian expression vector pcDNA 2.1/3.1, Western blots of transiently transfected SHSY5Y, a human neuroblastoma cell line, showed expression of two distinct bands representing the two isoforms (Fig 1D, left panel). In order to distinguish the variants from each other and wild type NCS-1, the NCS-1 variants were engineered to contain different tags (HA on NCS-1 Var1 and FLAG on NCS-1 Var2; Fig 1D, right panels). All six clones of stably transfected cell lines expressed the appropriate variant (shown as A1-6/B1-6; Fig 1D, right panel). These results show that human cell lines are capable of expressing both isoforms.

To compare the protein folding and calcium binding properties of the two NCS-1 variants, it was necessary to show that the proteins can be purified. NCS-1 Var1 and NCS-1 Var2 were expressed in BL21-CodonPlus (DE3)-RIL cells and purified with a hydrophobic interaction column (Fig 2A). Examination of the expressed proteins shows that the processed protein can be purified. A comparison of the protein concentration in each elution fraction shows that the profiles of the two variants are similar (Fig 2B). It also was necessary to show that the purified protein was stable for prolonged periods at room temperature so that biophysical assays could be performed in samples of protein lacking degradation. Over a 24 hour period while maintained at room temperature, 13 samples were obtained and examined for degradation. Both isoforms were stable for at least 12 hours (Fig 2C). However, there was some evidence of degradation of NCS-1 Var2 after 24 hours (right arrow in Fig 2C, lower panel).

### NCS-1 Var1 has a higher calcium binding affinity than NCS-1 Var2

**Fig 3:**
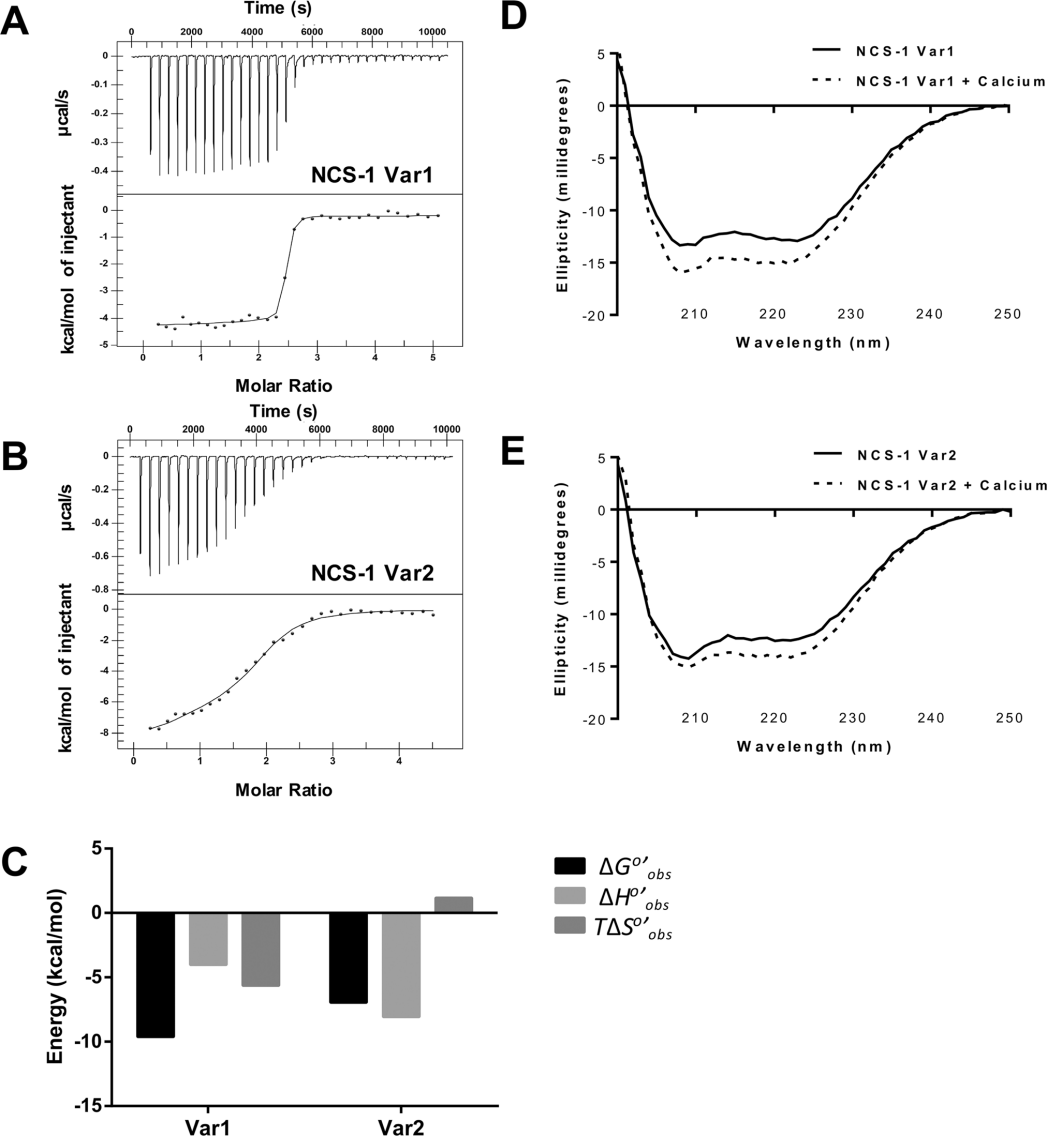
Calcium binding to NCS-1 variants assessed by ITC and CD. (A-B) The top plots of panels A and B depict the raw heat measurements of titrating 1.5 μL of 1.5 mM calcium into 100 μM NCS-1 Var1 and NCS-1 Var2, respectively, a result of isothermal titration calorimetry (ITC). The corresponding bottom plots show the fitted binding isotherms of the same experiment. (C) The graph shows the observed free energy change associated with calcium binding to each NCS-1 variant as well as their entropic and enthalpic contributions. The thermodynamic parameters derived from this experiment are listed in Table 1. (D-E) The effects of calcium binding on the conformational states of NCS-1 Var1 and NCS-1 Var2 are shown by the far UV circular dichroism spectra in panels D and E, respectively. The final protein concentration in each measurement was 2 μM, and the final calcium concentration in the ligand binding experiments was 600 μM. (F) The calcium binding induced changes in NCS-1 Var1 and NCS-1 Var2 secondary structure content in the far UV region from 200–240 nm was analyzed by *k2d* software from Dichroweb.

The calcium binding affinities of NCS-1 Var1 and NCS-1 Var2 were measured using ITC. Although the amino acid sequence between the two variants only differs at the N-terminus, and does not alter either the ancestral (non-functional) or the functional EF hands, the fitted binding isotherms reveal that the observed calcium binding affinity of NCS-1 Var1 (K_d_ = 96 nM) is nearly two orders of magnitude tighter than that of NCS-1 Var2 (8640 nM) (Fig 3A and 3B and Table 1). The number of effective binding sites for NCS-1 Var1 is calculated to be 2.4 whereas that of NCS-1 Var2 is 1.8 (Table 1). The small difference in number of calcium binding sites between Var1 and Var2 could reflect experimental uncertainty (e.g. protein concentration) or a reduction in functional Var 2 binding sites. The observed 2 orders of magnitude change in the observed calcium binding affinity strongly suggests that N-terminus modification of Var2 greatly weakens the average calcium affinity of each binding site. The different affinities are expected to affect the cellular calcium buffering capacity/properties of the variants.

**Table 1:** ITC Thermodynamic Parameters of Calcium Binding to NCS-1 Variants.

NCS-1	Dissociation constant, K_d_ (nM)	Number of Binding Sites, n	Enthalpy, Δ*H*°’_obs_ (kcal/mol)	Entropy Term, -*T*Δ*S*°’_obs_ (kcal/mol)	Gibbs Free Energy, *Δ*G°’_obs_ (kcal/mol)
Var1	96 ± 48	2.4 ± 0.02	-4.0 ± 0.1	-5.6	-9.6
Var2	8600 ± 3200	1.8 ± 0.05	-8.0 ± 0.6	1.1	-6.9

The thermodynamic parameters extracted from the experimental data reveal that calcium-binding to NCS-1 Var1 has comparable enthalpic (Δ*H*°′_obs_ = -4.0 kcal/mol) and entropic (-*T*Δ*S*°′_obs_ = -5.6 kcal/mol) contributions (Fig 3C and Table 1) to binding. We emphasize that Δ*H*°′_obs_ and *T*Δ*S*°′_obs_ represent observed, or overall, changes associated with calcium binding, which includes binding itself and any linked equilibria such as a conformational change and/or ion or proton release. In contrast, calcium-binding to NCS-1 Var2 is enthalpically driven (Δ*H*°′_obs_ = -8.0 kcal/mol,—*T*Δ*S*°′_obs_ = 1.1 kcal/mol; Fig 3C and Table 1).

These thermodynamic measurements indicate that the molecular origins of calcium binding to NCS-1 Var1 and Var2 differ under identical experimental conditions (e.g. temperature, salt concentration, and pH). The strong enthalpic contribution observed for NCS-1 Var2 suggests that calcium binding to NCS-1 Var2 is driven by bond formation (e.g. charge pair interactions, hydrogen bonding and/or van der Waals interactions); the small unfavorable entropy changes may reflect a modest reduction in conformational dynamics with calcium occupancy. In contrast, calcium binding to NCS-1 Var1 is associated with favorable enthalpic and entropic changes, reflecting stable bond formation and enhanced dynamics of NCS-1 and/or solvent.

To test if the N-terminal amino acid sequence differences caused a misfolding of NCS-1 Var2 that could account for the disparity in calcium binding affinities, we analyzed the secondary structure content of the two variants by measuring their far-UV circular dichroism spectra (Fig 3D and 3E). The spectra of NCS-1 Var1 and NCS-1 Var2 are sufficiently similar in the calcium-free states to suggest that NCS-1 Var2 is folded correctly and the spectra match those previously published [25]. We also compared the changes in the CD spectra induced by calcium binding and found that the addition of calcium shifts the CD spectra as expect for NCS-1 Var1 [25] but not for NCS-1 Var2. These differences in the CD spectra in the presence of calcium are different enough to support altered calcium binding, but remain similar enough to rule out misfolding of NCS-1 Var2.

### NCS-1 Var1 is the predominant transcript expressed in human cell lines

**Fig 4:**
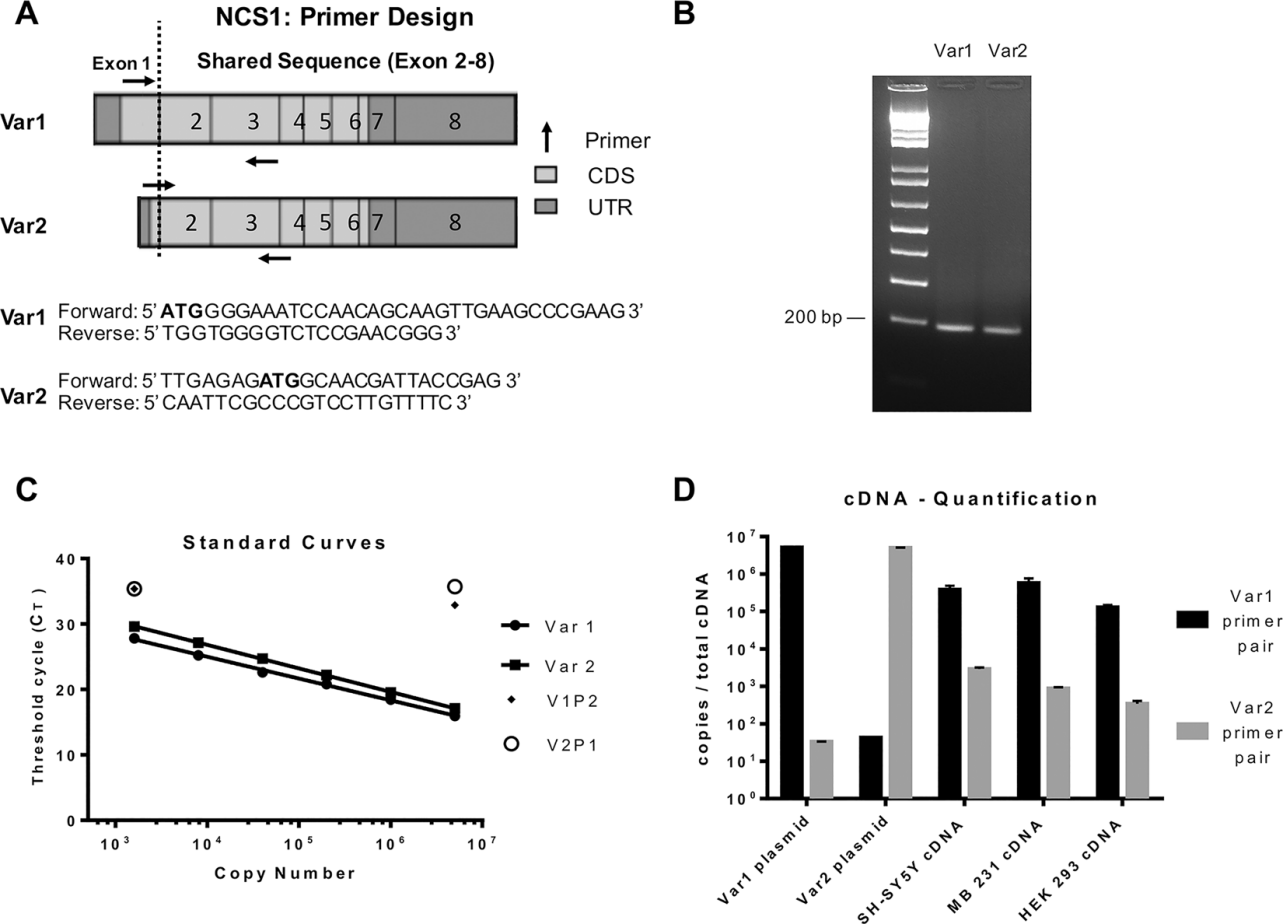
Quantification of NCS-1 variants cDNA. (A) Primer sequence and schematic primer alignment. In order to achieve the highest specificity for the reaction, primers for reverse transcribed mRNA of each NCS-1 isoform were designed according to the sequence of Exon 1. Based on the shortness of Exon 1 in NCS-1 Var2 (<20 bp) the forward primer overlaps with Exon 2. Both primers amplify a target region of approximately 200bp. (B) Agarose gel showing the amplification product of representative wells after RT-PCR. (C) Standard curves for NCS-1 Var1 and NCS-1 Var2. To analyze primer specificity the highest and lowest amounts of NCS-1 Var1 plasmid used in the standard curve were mismatched with NCS-1 Var2 primers (V1P2) and vice versa (V2P1). Note that in each case the primer mismatch cycle threshold (CT) value lies significantly above the highest CT value of either standard curve. (D) cDNA quantification of NCS-1 in SHSY5Y, HEK 293 & MB 231. Note that NCS-1 Var1 and NCS-1 Var2 differ in expression by approximately 3 log units. Each point was done in triplicate. The reverse transcribed mRNA (cDNA) was analyzed at two different concentrations where the figure shows results with a starting material of 5000 ng/well. Similar results were obtained with 200 ng/well. NCS-1 Var1 plasmid and NCS-1 Var2 plasmid serve as controls, the mismatch primer controls give a lower limit for interpretation, and the four bars on the far left show the maximum and minimum values for CT.

RT-PCR standard curve analysis showed that all three human cell lines tested, SHSY5Y, MB 231, and HEK 293, expressed transcripts of both *NCS1*-001 coding for NCS-1 Var1 and *NCS1*-003 coding for NCS-1 Var2. Because there is high sequence homology between the two transcripts, primer pair specificity was achieved through the distinctive sequence of exon 1 of the variants (Fig 4A). To rule out potential contamination through non-specific amplification of genomic DNA (gDNA), primers for NCS-1 Var2 were designed to overlap with the exon 1—exon 2 junction. A test for specificity included examination of the amplification products of both reaction types, that is an examination of the product from the NCS-1 Var1 primer pair and the NCS-1 Var2 primer pair (Fig 4B). To reach similar amplification efficiencies, the primers were designed to amplify a target region of approximately the same size, in this case 200bp. The resulting lanes show single sharp bands, indicating high specificity of each primer pair and clean amplification.

The relationship between the log of the copy number and the cycle threshold (C_T_) generated a linear trend. When we fit the data to a linear regression model, we calculated a goodness of fit of R-squared = (R^2^_Var1Std_ = 0.996; R^2^_Var2Std_ = 0.998), which allows quantification of the two transcripts (Fig 4C). To further demonstrate isoform specific amplification, the highest and lowest copy number of NCS-1 Var1 plasmid from the standard curve was mismatched with NCS-1 Var2 primers (V1P2) and vice versa (V2P1). In both cases the primer mismatch C_T_ value lies significantly above the highest CT value of either standard curve (Fig 4C). There is no difference in amplification in the mismatch test when comparing V1P2 and V2P1 at the two amounts of starting material (Fig 4C). In the test with either the high or low amounts of starting material of NCS-1 primers, the mismatched pairs have meaningfully greater CT values than the specific NCS-1 primer pairs. This difference is more pronounced at the higher copy number where the mismatch pairs are separated at least 20 cycles from the specific pairs. These results provide further support of the specificity of the primers and allows quantitative comparisons of the two variants of NCS-1.

To determine the relative amounts of mRNA of the two NCS-1 variants we compared the amount of mRNA of NCS-1 Var1 and NCS-1 Var2 in three human cell lines. Mismatched primer pairs with high (and low, not shown) starting amount of plasmid were used to determine a lower limit for the expression of the variants (Fig 4D, 2nd and 3rd bars from left) and matching primer pairs, the ideal amplification conditions, were used to determine maximum expected expression (Fig 4D, 1st and 4th bars from left). We would have been able to specifically detect mRNA expression at a concentration of 100 copies/well (with a reaction volume of 20uL) This estimate is obtained from our mismatch primer control where the highest and lowest concentrations of template (5,000,000 copies/well and 1,600 copies/well, respectively) were primer-mismatched and amplified on the same plate as the rest of the experiment. The threshold cycle for these wells is approximately 40 (Fig 4C), a value usually associated with background levels, and this value does not change significantly with template starting amount; this result is the opposite for the (non-mismatched) standard curve. For each cell line tested, the NCS-1 Var2 mRNA levels were approximately 1000 fold lower than NCS-1 Var1 transcripts (Fig 4D, left bars), showing that NCS-1 Var1 is the predominant transcript in the cells tested.

### NCS-1 Var2 protein was not detected in human cell lines

**Fig 5:**
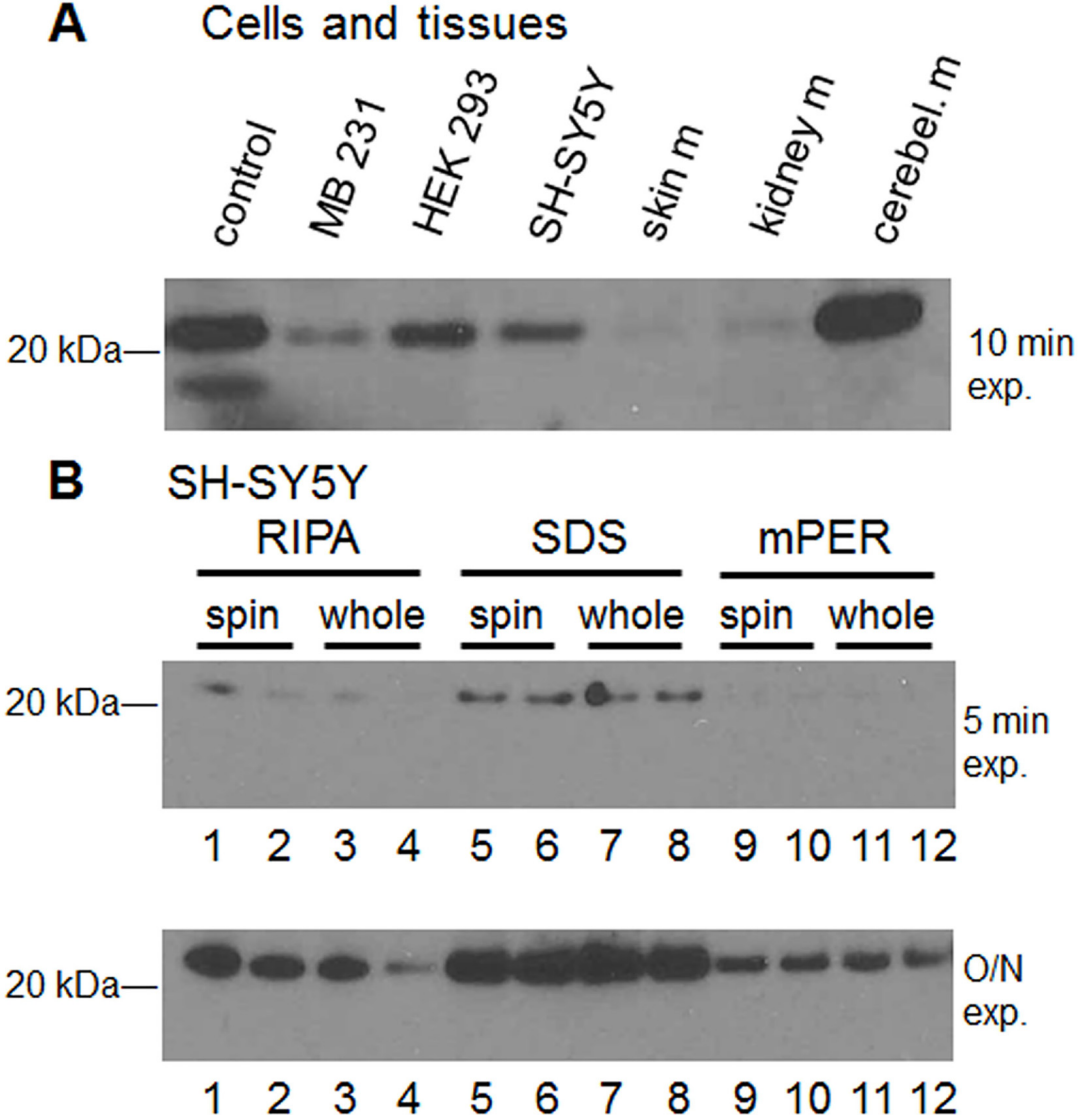
Expression of NCS-1 variants in cell lines. (A) Representative Western blot showing the expression of NCS-1 Var1 in different human cell lines (SHSY5Y, HEK293, MB231) and C57BL/6 mouse tissues using a polyclonal anti-NCS-1 antibody (FL190 Santa Cruz). As a control, known amounts of purified NCS-1 Var1 and NCS-1 Var2 protein were used in Lane 1. Each lane was loaded with 35 μg of protein. Note that only NCS-1 Var1 is identified in all tissue lanes. (B) To show that lysis conditions were not responsible for loss of NCS-1 Var2, three lysis buffers (RIPA, SDS, mPER), sample preparations (spun down supernatant (spin) or whole cell lysate (whole)) samples were either boiled at 95°C for 10 mins before loading (odd numbered lanes) or loaded without boiling (even numbered lanes), and film exposure times (5 min or overnight (O/N)) were compared. Regardless, NCS-1 Var2 was not identified in SHSY5Y5 cells.

To determine whether the difference in mRNA amount of the two variants reflected the amount of each variant of NCS-1 protein expressed, Western Blot analysis was done. As a control, known amounts of purified NCS-1 Var1 and NCS-1 Var2 protein were included (Fig 5A, left most lane). This test shows that the anti NCS-1-antibody is able to detect both isoforms (Fig 5A). The lack of NCS-1 Var2 in mouse tissues (Fig 5A, 3 right lanes) was expected from examination of the online databases (National Center for Biotechnology Information (NCBI) [22] and Ensembl genome browser 83 (doi: 10.1093/nar/gkt1196)). which indicate the existence of a second isoform transcript only in humans (whereas mouse tissue only contain a sequence that is thought to undergo nonsense mediated decay) However, despite inclusion of mRNA for both variants in human cell lines, only NCS-1 Var1 is identified in all native cell and tissues tested (Fig 5A, lanes 2–4). As expected, the expression of NCS-1 Var1 in neuronal tissue (as indicated by the dark bands of cerebellar mouse tissue and SHSY5Y cell lysate) is higher than in other tissue types.

To show that lysis conditions were not responsible for loss of NCS-1 Var2, three lysis buffers (RIPA, SDS, mPER), sample preparations (spun down supernatant (spin) or whole cell lysate (whole), boiled (odd numbered lane) or unboiled (even numbered lane)) were compared (Fig 5B top). In addition, two different film exposure times (5 min or overnight (O/N)) were compared(Fig 5B bottom). Regardless of the conditions, NCS-1 Var2 was not identified in SHSY5Y5 cells.

### Over-expression of either NCS-1 Var1 or Var2 does not alter cell growth or death

**Fig 6:**
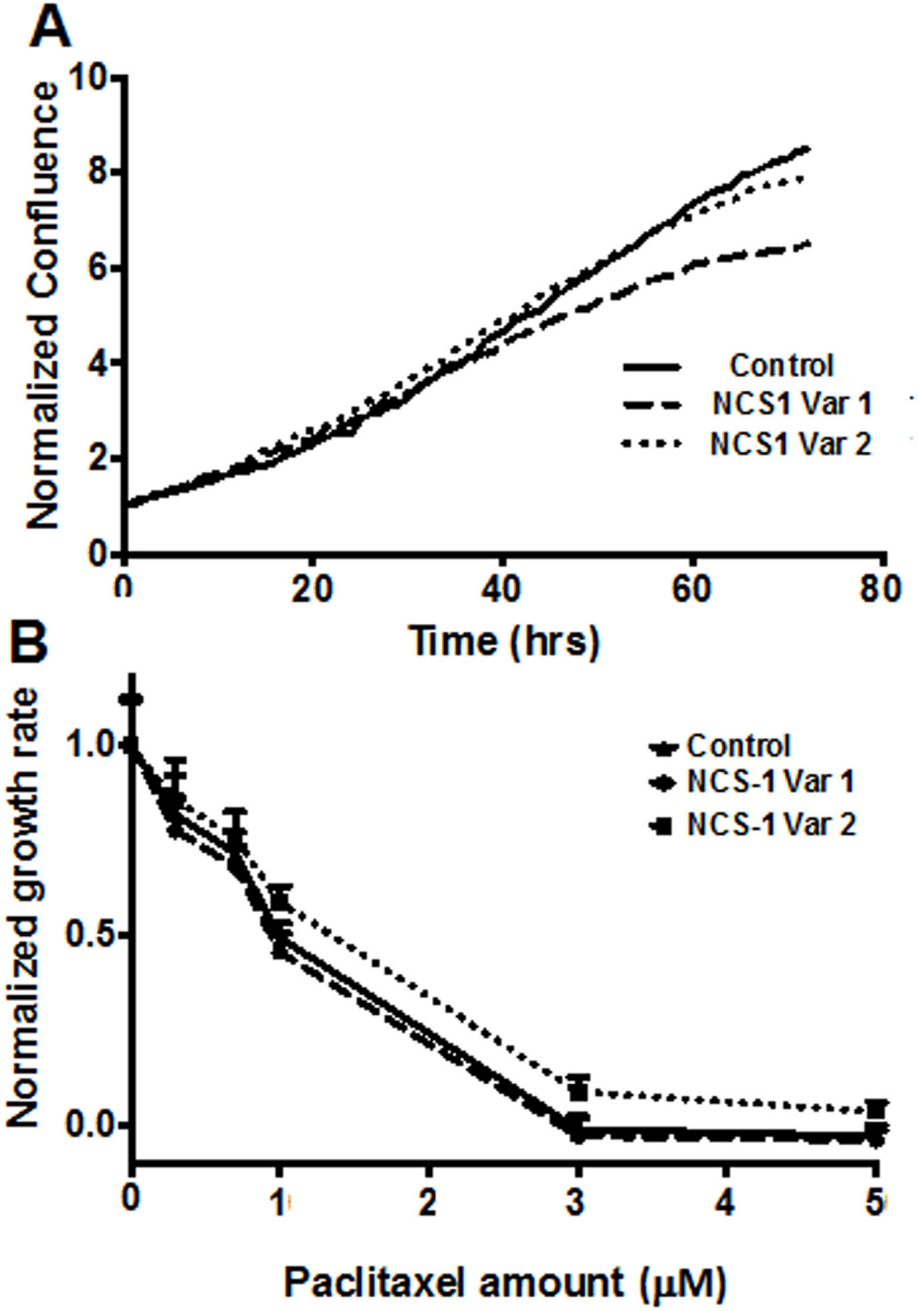
Effect of NCS-1 variants expression on proliferation and sensitivity to paclitaxel. (A) Cell proliferation was monitored in cells stably expressing NCS-1 Var1, NCS-1 Var2, or a scrambled vector. (B) The concentration dependence of paclitaxel induced cell death. Note that expression of NCS-1 variants did not alter the effect of paclitaxel.

Because NCS-1 Var2 binds calcium with a much lower affinity compared to NCS-1 Var1, functional differences were expected when comparing cells expressing the two variants. Calcium signaling affects cell growth rate and cell death by regulating a number of cell pathways [26, 27]. To determine if NCS-1 Var1 and NCS-1 Var2 differentially affect cell growth rate, we generated SHSY5Y cells stably transfected with NCS-1 Var1, NCS-1 Var2, or an empty vector used as a control. Images were taken every hour to monitor cell confluence. We found that the cell growth rate was the same when comparing the cells stably over-expressing either variant or the vector (Fig 6A). This result suggests that NCS-1 does not, or the intracellular NCS-1 concentration is not sufficient, to alter the intracellular calcium required for cell growth.

We previously showed that NCS-1 Var1 binds the chemotherapeutic drug, paclitaxel, and this binding appears to be responsible for increased calcium release from the endoplasmic reticulum [20]. To test whether cells stably transfected with NCS-1 Var1, NCS-1 Var2, or an empty vector control responded differentially to the addition of paclitaxel, the rate of cell death after addition of increasing concentrations of paclitaxel was monitored (Fig 6B). The loss of cells was the same in all the stably transfected cells (Fig 6B). Again, this result shows that amount of the overexpressed NCS-1 variants does not change the cell’s response to the addition of paclitaxel over the range of concentrations tested.

## Discussion

The aim of this study was to determine if the variants of NCS-1 were functionally important in regulating intracellular calcium signaling. Alterations in NCS-1 function have been implicated in a number of human diseases including schizophrenia [12], bipolar disease [13] and chemotherapy-induced peripheral neuropathy [15]. These previous reports have implied that changes in the levels of NCS-1 Var1, and subsequent changes in calcium transients, were responsible for the alterations in cell function. However, the possible expression and role for variants of NCS-1 had not been examined and the previous reports did not distinguish among variants of NCS-1. We hypothesized that NCS-1 Var2 had a functional role and that the expression level was relevant to human tissues.

Here we show that an under-studied variant of NCS-1 with a large alteration to the N-terminal amino acids (NCS-1 Var2), where 22 amino acids are replaced by 4 different amino acids, has a mRNA expression level 1000 times lower than that of NCS-1 Var1. Moreover, NCS-1 Var2 has almost 100 times lower affinity for calcium. This change in calcium affinity occurs even though the protein folding, as measured by CD, appears similar in the two variants. The high calcium affinity and much higher mRNA expression level of NCS-1 Var1 in the cell compared to NCS-1 Var2 suggests that NCS-1 Var1 will be the functionally dominate variant.

The first step in examining the biochemical properties of the variants of NCS-1 was to quantify mRNA levels in three cell lines (SHSY5Y, HEK 293 and MB 231). We verified that NCS-1 Var1 and NCS-1 Var2 exist in these human cell lines, but the ratio of the expression of the two variant differed by approximately 3 log units. Even though the three different cell lines expressed different absolute amounts of NCS-1 mRNA, all cell lines showed a ratio of approximately 1000:1(Var1: Var2). This result indicates that NCS-1 Var1 mRNA is the dominant version found in cells.

Because mRNA levels do not always directly correlate with protein expression levels, the second step in examining the properties of the NCS-1 variants was to monitor the protein levels by Western Blot analysis. We were not able to detect NCS-1 Var2 in SHSY5Y, HEK293, MB231 cell lines. These cell lines were selected because they are all derived from human tissue. We also examined mouse tissues even though the mRNA for the NCS-1 variants have only been previously identified in human tissues. NCS-1 Var2 was not detected in mouse skin, kidney, cerebellum, as expected. If the protein levels reflect the ratio of mRNA, a ratio of 1000:1 for the protein could be predicted and this level of NCS-1 Var2 may be outside the detection limit. However, after a large number of conditions were used to look for the second variant (see Fig 5), we conclude that NCS-1 Var2 is either not expressed or expressed in amounts too low to detect with the tools available in cell lines or tissue.

Despite the lack of expression of NCS-1 Var2 in quiescent mammalian cells, understanding the properties of NCS-1 Var2 is interesting because it has properties intermediate between NSC-1 Var1 (the native protein) and the version of NCS-1 that is cleaved by calpain [9]. NCS-1 has several cleavage sites in the N-terminal region and when calpain is allowed to cut to completion, the N-terminal 36 amino acids of NCS-1 are lost, leaving a truncated protein that does not bind calcium [9]. When calpain is allowed to cleave NCS-1 for short periods, cuts that are upstream of the site at residue 36 occur. NCS-1 Var2 lacks the first 18 N-terminal amino acids, which is approximately half of the first helix of the protein and would be a mimic of a transient intermediate protein that is the product when calpain cleaves NCS-1 Var1, as occurs when paclitaxel is administered. The helix at the N-terminus of NCS-1 appears to form a cap that protects the proximal calcium binding site. When this site is fully exposed, the affinity to calcium binding sites drops [9], likely because removal of the first helix perturbs the second EF hand [9]. Our results show that even though NCS-1 Var2 maintains only a portion of the cap, it is still able to bind calcium, but at a much lower affinity, suggesting that removal of only a portion of the cap can still perturb the second EF hand. If calpain is activated only transiently, as might occur in during chemotherapy with paclitaxel, a compound that has been shown to lead to the activation of calpain [18] [21] the intermediate versions of NCS-1, similar to NCS-1 Var2, would exist in the cell. However, the lower affinity of this version of NCS-1 would make it less effective as a regulatory agent for calcium [27].

The next step in the examination of NCS-1 variants was to ask whether the differing properties of the NCS-1 variants could be responsible for the altered cell function. The observation that NCS-1 protein is cleaved by calpain during chemotherapy raised the question whether this variant could be a linked to the pathophysiology associated with chemotherapy. Because we found that NCS-1 Var2 binds calcium with an affinity that is two orders of magnitude lower than NCS-1 Var1, we expected to observe detectable functional changes when comparing cells expressing the two variants. However, the rate and extent of cell proliferation and the ability of paclitaxel to induce cell death were indistinguishable when comparing cells expressing the two variants of NCS-1. These results suggest that overexpression of either variants of NCS-1 does not alter cell proliferation. Paclitaxel administration induces cell death by stabilizing microtubules [28]. overexpression of either variant of NCS-1 could have diminished the effectiveness of paclitaxel to induce cell death, in part by overwhelming the cell with an alternative binding partner. However, our experiments showed that overexpression of NCS-1 does not impair this effect of paclitaxel.

NCS-1 regulates a number of cell pathways by binding to protein partners and this binding usually occurs using the hydrophobic pocket identified in the structure of NCS-1 [27, 29]. Because the crystal structure [29, 30] shows that the hydrophobic pocket and the first helix are positioned on opposite surfaces of the protein, the removal of the first helix should have little effect on the hydrophobic ligand binding pocket. One difference in the NCS-1 variants could have been the degree of exposure of the hydrophobic pocket of NCS-1 after addition of calcium because calcium binding can induce a structural change [25, 31]. Because we used a hydrophobic affinity column to purify the protein, a comparison of the protein concentration profile in the fractions eluted from the column gave insight into this parameter. The elution profiles of the two variants of NCS-1 are similar, supporting the idea that NCS-1 Var2 has similar exposure of the hydrophobic pocket. Because this hydrophobic pocket is intact, NCS-1 Var2 still has the capacity to bind, and possibly influence, protein partners, especially those that are calcium independent. However, until conditions are identified where the expression of NCS-1 Var2 is comparable to the regulated protein partner, it seems unlikely that NCS-1 Var2 has critical functions in cells.

In conclusion, we have examined a variant of NCS-1 that was identified in the human transcriptome. Although mRNA for NCS-1 Var2 can be detected, albeit at a low level, protein expression was not detectable. When overexpressed in cell lines NCS-1 Var2 appears to be folded properly, yet it has a lower calcium affinity. Because most NCS-1 binding to its protein partners requires calcium binding [27] it is unlikely that NCS-1 Var2 would play a major regulatory role in cells even if its expression level were amplified to be on a par with NCS-1 Var1. These results suggest that it is indeed the alterations of the levels of NCS-1 Var1 expression that is critical for human disease.

## Abbreviations

bp: base pair
CDS: coding sequence
*K*_d_: dissociation constant
Δ*H*°′_obs_: enthalpy
Δ*S*°′_obs_: entropy
G418: Geneticin
gDNA: genomic DNA
HIC: hydrophobic interaction chromatography
InsP3R: inositol trisphosphate receptor
IPTG: isopropyl-d-thiogalactoside
NCBI: National Center for Biotechnology Information
NCS-1: Neuronal calcium sensor-1
NCS-1 Var1: Neuronal calcium sensor-1 Wild type
NCS-1 Var2: Neuronal calcium sensor-1 Variant 2
TCEP: *tris*(2-carboxyethyl)phosphine
